# Implementation Strategies to Enhance Youth-Friendly Sexual and Reproductive Health Services in Sub-Saharan Africa: A Systematic Review

**DOI:** 10.3389/frph.2021.684081

**Published:** 2021-08-04

**Authors:** Chisom Obiezu-Umeh, Ucheoma Nwaozuru, Stacey Mason, Titilola Gbaja-Biamila, David Oladele, Oliver Ezechi, Juliet Iwelunmor

**Affiliations:** ^1^College for Public Health and Social Justice, Saint Louis University, Saint Louis, MO, United States; ^2^Clinical Sciences Department, Nigerian Institute of Medical Research, Lagos, Nigeria

**Keywords:** adolescent, young people, youth-friendly health services, sexual and reproductive health, implementation science, implementation strategies, implementation outcomes, sub-Saharan Africa

## Abstract

**Introduction:** Youth-friendly health service (YFHS) interventions are a promising, cost-effective approaches to delivering sexual and reproductive services that cater to the developmental needs of young people. Despite a growing evidence-base, implementation of such interventions into practice have proven to be challenging in sub-Saharan Africa (SSA). Thus, the purpose of this review is to synthesize existing evidence on YFHS implementation in SSA and understand which implementation strategies were used, in what context, how they were used, and leading to which implementation outcomes.

**Methods:** A comprehensive literature search in PubMed, Embase, Scopus, and CINAHL, was conducted to identify peer-reviewed research articles published from database inception up until August 2020. Eligible studies were required to include young people (ages 10–24 years) in sub-Saharan Africa. Studies that described implementation strategies, as conceptualized by the Expert Recommendations for Implementing Change (ERIC) project, used to enhance the implementation of YFHS were included. Implementation outcomes were extracted using Proctor and colleagues' 8 taxonomy of implementation outcomes.

**Results:** We identified 18 unique interventions (reported in 23 articles) from an initial search of 630 articles, including seven from East Africa, seven from South Africa, and four from West Africa. In most studies (*n* = 15), youth-friendly health services were delivered within the context of a health facility or clinic setting. The most frequently reported categories of implementation strategies were to train and educate stakeholders (*n* = 16) followed by infrastructure change (*n* = 10), to engage consumers (*n* = 9), the use of evaluative and iterative strategies (*n* = 8), support clinicians (*n* = 8), and providing interactive assistance (*n* = 6). The effectiveness of the strategies to enhance YFHS implementation was commonly measured using adoption (*n* = 15), fidelity (*n* = 7), acceptability (*n* = 5), and penetration (*n* = 5). Few studies reported on sustainability (*n* = 2), appropriateness (*n* = 1), implementation cost (*n* = 1) and feasibility (*n* = 0).

**Conclusion:** Results of the review emphasize the need for further research to evaluate and optimize implementation strategies for promoting the scale-up and sustainability of evidence-based, YFHS interventions in resource-constrained settings. This review also highlights the need to design robust studies to better understand which, in what combination, and in what context, can implementation strategies be used to effectively enhance the implementation of YFHS interventions.

## Background

Across sub-Saharan Africa (SSA), youth (aged 10–24) have high unmet need for sexual and reproductive health (SRH) and the existing SRH services may not have the capacity to fully address their developmental needs (1, 2). As a result, utilization of SRH preventive and treatment services among youth remains low (3–5). This is partly explained by multiple barriers in accessing SRH services including lack of awareness on where to get services, fear of lack of confidentiality and privacy, parental consent, cost of services, distance, and negative provider attitudes (6). Furthermore, existing SRH services are configured for adults and often served by adults who are not sensitive to their needs (7, 8). The inadequacy in SRH service provision and utilization in this region is congruent with high rates of sexually transmitted infections (STIs), including HIV, unsafe abortion, early and unintended pregnancies among youth (8, 9). Of even greater concern is that more than half of new HIV infections in SSA still occur among those aged 15–24 years (10) and adolescent girls and young women are 14 times more likely to be newly infected with HIV than their male counterparts (11). The prevalence of STIs is also high among this population, such that a third of the 333 million estimated cases of curable STIs (gonorrhea, syphilis, chlamydia, and trichomoniasis) are reported annually among individuals <25 years old, followed by individuals between the ages of 15–19 years (12).

Youth-friendly health services (YFHS) are one of the evidence-based interventions recommended by the World Health Organization (WHO) to address health system barriers by providing health-enabling social environments that are more accessible, acceptable, equitable, appropriate and effective for young people (13). Such services vary by type of care provided and cover a range of services and commodities including counseling and referral for contraceptives and condoms, education on sexual and reproductive health, HIV counseling and testing, and STI screening and treatment (14, 15). Despite established effectiveness and implementation efforts, such interventions are not rapidly scaled-up or sustained over an extended period after external support is terminated, delaying or halting benefits to end-users and health systems (16–19). Much uncertainty still exists about the ideal service delivery strategies that are sensitive to their sexual reproductive health needs.

Implementation strategies, defined as “approaches or techniques used to facilitate the adoption, implementation, sustainment, and scale-up of evidence-based health innovations into usual care” (20, 21), may be used to enhance the implementation of YFHS interventions into practice. The Expert Recommendations for Implementing Change (ERIC) project (22) suggests a taxonomy for organizing 73 discrete implementation strategies into nine overarching categories (i.e., engage Consumer, change infrastructure, train and educate) (Table 1) that have been successfully used by other researchers (24–27). Although research on implementation strategies is still in its infancy, there has been an increasing recognition that passive implementation efforts are not enough to narrow the 17-years research-to-practice gap (28). Thus, the use of implementation strategies could be effective in improving processes and outcomes, especially when tailored to different implementation contexts (21, 29, 30). The degree in which implementation strategies have been successfully utilized can be evaluated on the basis of implementation outcomes (23). Research literature has summarized different aspects of YFHS implementation for improving SRH outcomes (5, 6, 31, 32), including barriers to provision and use of YFHS (19, 33) and assessment of YFHS (34–36). However, evidence regarding the effective use of strategies in the implementation of youth-friendly sexual and reproductive health services, has not yet been summarized or reviewed. Additionally, the relationship between the implementation strategies and implementation outcomes, is rarely highlighted. Thus, this study expands on previous literature to synthesize the evidence regarding (1) which implementation strategies were used while implementing the youth-friendly sexual and reproductive health interventions; and (2) which implementation outcomes were achieved.

**Table 1:** Implementation Strategies and implementation outcomes.

Implementation strategies^*^	**Description**
Engage consumer	Involving, intervening and preparing with young people and community members; increasing demand for services.
Use evaluative & iterative strategies	Assessing readiness for implementation and conducting needs assessments; developing implementation plans; evaluating performance and progress; providing audits and feedbacks; developing and implementing quality monitoring tools.
Change infrastructure	Changing physical structures/locations of clinic/services, as well as internal conditions such as equipment.
Adapt & tailor to the context	Tailoring intervention/services to meet local needs.
Develop stakeholder interrelationships	Identifying and preparing implementation teams; organizing preparatory meetings and convening advisory committee; identifying and recruiting key opinion leaders and change agents that will support and help drive implementation.
Utilize financial strategies	Creating and utilizing fee structures and incentives.
Support clinicians	Creating clinical teams and revising professional roles.
Provide interactive assistance	Providing technical assistance and supportive supervision to enhance clinical performance.
Train & educate stakeholders	Conducting trainings and providing educational opportunities; creating a learning environment.
Implementation outcomes^*^	**Description**
Acceptability	Perception among youths and other stakeholders that a given treatment, service, practice, or innovation is agreeable, palatable, or satisfactory.
Adoption	Intention, initial decision, or action to try or employ an innovation or evidence-based practice.
Appropriateness	Perceived fit, relevance, or compatibility of the innovation or evidence-based practice setting, provider, or consumer; and/or perceived fit of innovation to address an issue.
Feasibility	Extent to which a new treatment, or an innovation, can be successfully used or carried out within a given agency or setting.
Fidelity	Degree to which an intervention was implemented as it was prescribed in the original protocol or intended by the program developers; level of exposure, dose or amount and quality of the intervention.
Costs	Cost impact of an implementation effort (i.e., implementation costs).
Penetration	Integration of a practice within a service setting and its subsystems.
Sustainability	Extent to which a newly implemented treatment is maintained or institutionalized within a service setting's ongoing, stable operations.

* *Definitions for implementation strategies and outcomes were adapted from Waltz et al. (22) and Proctor et al. (23), respectively*.

## Methods

We conducted a systematic review of peer-reviewed, published studies using pre-defined implementation science concepts to understand which implementation strategies were used, in what context, how they were used, and which implementation outcomes were achieved. Table 1 describes the definitions and categorizations of the implementation outcomes and strategies used in this study.

### Search Strategy

**Figure 1:**
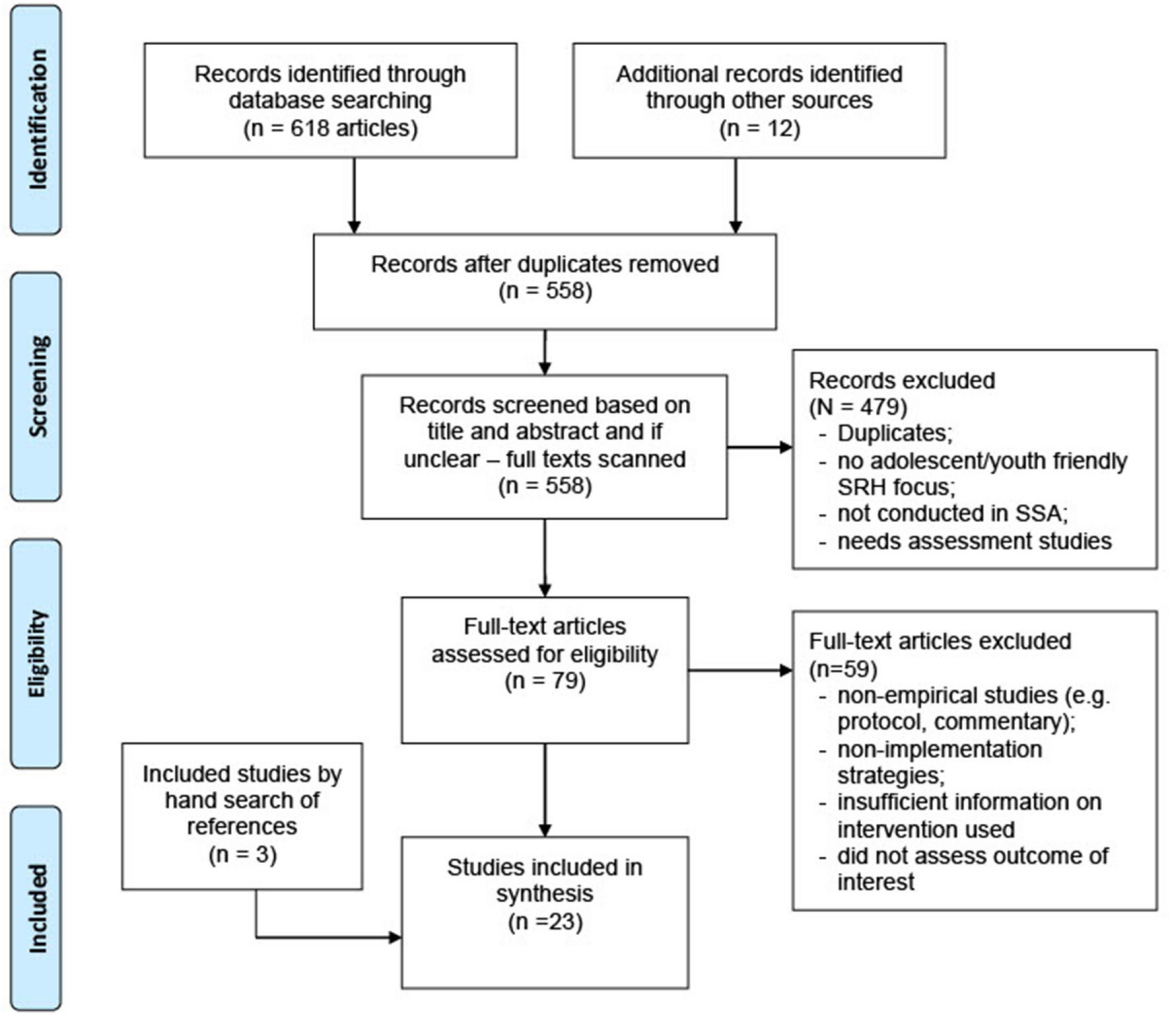
PRISMA flow diagram.

Figure 1 outlines the search strategy, which was reported in accordance with the Preferred Reporting Items for Systematic Reviews and Meta-Analyses (PRISMA) statement and checklist. With the guidance of a medical librarian, a comprehensive search strategy was devised using a combination of subject heading terms and keywords for youth-friendly or adolescent-friendly, health services or clinics and sub-Saharan Africa. The search was conducted in four electronic databases, including PubMed/Medline, Embase, CINAHL and Global Health, from database inception to October 12th, 2019. An updated search was also conducted in August 20th, 2020. Bibliographies or reference list of all identified articles were reviewed manually for additional studies.

### Eligibility Criteria

All identified review articles were assessed against the inclusion and exclusion criteria outlined in Table 2. These relate to the study location (where the study was conducted), the population of interest (beneficiaries of the intervention), intervention (what strategy was used and in what setting), outcomes (impact of the intervention i.e., SRH outcomes) and evaluation design (i.e., RCT or quasi-experimental study). Studies were included if implementation strategies were used when implementing the youth-friendly sexual and reproductive health interventions in a sub-Saharan Africa region. Empirical studies written in English in peer-reviewed journals were included. Non-empirical articles (i.e., reviews, editorials, commentaries), non-peer-reviewed articles, and studies focused on other specialty care such as mental health, were excluded.

**Table 2:** Inclusion and exclusion criteria.

**Inclusion criteria**	**Exclusion criteria**
**Setting**	
Study or evaluation conducted in a sub-Saharan Africa region.	Study or evaluation conducted outside sub-Saharan Africa.
**Population**	
Services or intervention serving clients aged 10–24 years.	Study population >24 years of age or not clearly described.
**Intervention**	
Provided detailed description of the implementation strategies used.	Insufficient description of the implementation strategy used.
Included sufficient description of youth-friendly SRH service intervention. Young people receiving SRH services within a facility (clinics, hospital, social franchise network), out-of-facility (schools, outreach/community), or youth centers.	Insufficient description of the intervention and its implementation.
**Outcomes**	
Utilization or distribution of SRH services or clinical SRH outcome (e.g., prevalence of HIV and other STIs).	Studies focused on other specialty care such as mental health. Quantitative assessment of measures of service utilization without assessment of change among young people over time or in comparison with the control group.
**Evaluation design**	
Randomized controlled trials; Quasi-experimental designs; Before/after comparison (without control group); Cross-sectional comparison to unexposed group or presented by level of exposure.	Interventions that did not utilize designs that enabled the evaluation of the impact of an intervention or inferences based on statistical tests.

### Article Selection, Data Extraction, and Analysis

The references were organized and screened using EndNote X8. Two authors (CO and UN) independently reviewed and selected eligible articles based on the predefined selection criteria in Table 2. The authors scanned through the titles and abstracts of the articles to exclude duplicates and studies that were not relevant to the topic of interest. Articles were selected for full-text review upon agreement by both authors. Following the full-text review, the first author and another co-author independently reviewed the full-text articles. The authors met severally during this process to reach an agreement where discrepancies arose and ensure understanding of the inclusion and exclusion criteria.

Data were extracted using a piloted data extraction tool, developed by the authors, relating to study details, country of origin, study population, setting, service delivery model, implementation strategy employed, implementation science outcome achieved, research question; and key study findings. Service delivery models were classified according to Simon et al. (37) definitions: Model 1-Standalone clinic (completely separate health center/clinic dedicated to serving youths); Model 2-Separate space for YFHS (separate spaces co-located in public or private health facilities); Model 3-Mainstreamed YFHS (integrated/mainstreamed within public or private health facility/not a separate space); Model 4-Mobile outreach services (services offered in non-routine sites or by a mobile team of health providers at lower-level health facilities); Model 5-Community-based services (offered through community-based outreach services by peers or community health workers); Model 6-Servies offered within Drug shops and pharmacies; Model 7-SRH services in non-health settings/informal settings.

The implementation strategies were sorted according to the nine categories described by the Expert Recommendations for Implementing Change (ERIC) project (see Table 1) (22). The identified implementation outcomes were categorized based on Proctor et al. eight taxonomy of implementation outcomes: acceptability, adoption, appropriateness, feasibility, fidelity, costs, penetration, and sustainability (see Table 1) (23). Traditional quality assessment of the eligible studies was not conducted. Only studies that directly answered the research questions of the review were considered. Rigor was determined based on the authors' credibility of the conclusions made in the included studies.

## Results

### Overview of Included Studies

The searches resulted in 630 articles from online databases. After removing the duplicates, 558 unique titles remained. A total of 23 articles describing 18 unique interventions in SSA, were identified as relevant and eligible for inclusion in the review. In addition, reference indexing yielded three studies from the first 20 included studies (refer to Figure 1 for PRISMA flow diagram).

Of the 18 intervention studies, 33.3% (6/18) were randomized control trials; 22.2% (4/18) and 11.1% (2/18) were quasi-experimental studies with and without comparisons, respectively; and 33.3% (6/18) were other quantitative study designs. Majority of the studies were published between years 2015 and 2020. Studies were well-represented in most regions of sub-Saharan Africa with 38.9% (7/18) of the studies conducted in East Africa, 38.9% (7/18) in South African, and 22.2% (4/18) in West Africa. Specifically, the studies were conducted in 11 of the 46 SSA countries: Ghana (*n* = 1), Zimbabwe (*n* = 2), Ethiopia (*n* = 1), Uganda (*n* = 2), Kenya (*n* = 2), Tanzania (*n* = 2), South Africa (*n* = 2), Zambia (*n* = 1), Malawi (*n* = 2), Nigeria (*n* = 2), and Togo (*n* = 1).

Sixteen of the 18 intervention studies evaluated the effectiveness of adolescent- and youth-friendly interventions on health service utilization for HIV and other STI testing (38, 43–45, 51, 53, 55, 57, 58) or contraception use (i.e., condoms, long-acting reversible contraceptive) (40, 42, 51, 52, 54, 56, 57, 59). Whereas, two studies tested the impact of adolescent- and youth-friendly interventions on clinical SRH outcomes (i.e., rates of HIV and other STIs) (39, 45), and one study focused on the effect of YFHS on knowledge, attitudes, and screening behaviors among young people (60). Further characteristics of the included studies can be found in Supplementary Table 1.

### Models of Service Delivery

Using Simon et al. (37) definitions of models for delivering YFHS, interventions were categorized in ways that best represented the variety of strategies used in the studies. Eleven of the 18 studies utilized a combination of two different service delivery models (38–40, 42–45, 52, 53, 56, 60). Of the 18 intervention studies, 15 reported to have delivered YFHS within the context of a health facility or clinic setting (model 1, 2, or 3). Whereas, three studies in South Africa and Togo reported to have delivered services solely outside of the health facility/clinics (model 4, 5, 6, or 7) with the goal of taking the services to where young people congregate or leave (50, 58, 59). There was no clear pattern in trends in the model of service delivery and country of implementation.

### Implementation Strategies

#### Overview

All nine categories of the ERIC classification of implementation strategies were reported in the included studies. Three of the 18 studies (40, 54, 55) reported implementation strategies within 5 or 6 categories, thirteen (38, 39, 42–45, 49, 51, 52, 56, 57, 59, 60) reported implementation strategies within 3 or 4 categories and two (53, 58) reported implementation strategies in 1 or 2 categories. The most frequently reported implementation strategies were to train and educate stakeholders (*n* = 16, 88.9%) followed by change infrastructure (*n* = 10, 55.6%), engage consumers (*n* = 9, 50.0%), use evaluative and iterative strategies (*n* = 8, 44.4%), support clinicians (*n* = 8, 44.4%), and provide interactive assistance (*n* = 6, 33.3%). The least reported implementation strategies were to develop stakeholder interrelationships (*n* = 5, 27.8%), adapt and tailor to the context (*n* = 4, 22.2%) and use financial strategies (*n* = 1, 5.6%).

#### Engage Consumers

Nine of the 18 studies reported various strategies for engaging young people in the implementation of YFHS interventions. This included promoting SRH information to increase knowledge and use of SRH services through a mix of mass media campaigns and communication channels, such as posters, leaflets, newsletters, radio programs, dramas, and community campaign events (42, 43, 53, 54). Other implementation strategies reported engaging young people as peer-support workers (i.e., peer educators) to relay SRH information and provide services to other youths (39, 43, 45–48, 53), participants in an open dialogue with health providers to discuss their needs and preferences (38, 54) or participants in program design at the clinic level (59). One study also involved young people in the selection of the youth-friendly health providers (including patent medicine dealers and pharmacists) that served a considerable number of young people in the community (56).

#### Use Evaluative and Iterative Strategies

Eight studies reported the use of evaluative and iterative strategies that were mostly deployed in combination with other strategies during the pre-implementation, implementation, and post-implementation phases. Three studies reported assessing the health facility readiness, barriers, and facilitators before (38, 55) or after the implementation of the YFHS intervention (41). Whereas, five studies (39, 44, 52, 54, 55) developed a quality monitoring system to ensure that the YFHS intervention was implemented appropriately, with one (39) of these also providing continued auditing of adolescent- and youth-friendly service standards. One study (52) utilized a feedback mechanism whereby patients provided feedback on overall experience following each youth-friendly clinic visit and one study (60) implemented weekly team meetings to discuss change concepts, review clinic processes, and improvements.

#### Change Infrastructure

Ten studies employed a change in human and/or physical infrastructure to improve services for young people within the health facility or community-based setting. This included implementing extended hours within the clinics (44, 57), integrating youth-friendly health services as part of routine service delivery, and establishing social franchise networks within clinics (42–44, 55, 57). Community-based services included establishing health clubs within schools to educate on SRH and facilitate referrals to clinics (49, 50, 56), mobile health clinics for youths (58), and SRH services within youth centers (59) to reach more young people through the promotion of recreational activities.

#### Adapt and Tailor to the Context

Four studies reported having adapted the YFHS implementation guideline before full implementation of the study to meet the local needs and organizational capabilities (39, 49, 50, 52). For instance, in a study conducted in Zimbabwe, the intervention curriculum that was initially developed in Tanzania was adapted to the Zimbabwean cultural educational context (61).

#### Develop Stakeholder Interrelationships

Five studies reported the use of strategies for developing relationships among multiple key stakeholders. Implementation team meetings were organized with representatives of participating organizations, community members and youth, to build community buy-in and ownership (38, 55). Onsite leadership teams were formed within the clinics to support intervention in two studies (42, 60). One study established a coalition between schools and youth-friendly clinics to increase access to SRH information and services (52).

#### Use Financial Strategies

One study reported to have used conditional cash transfers among participants who successfully completed a series of small, interactive group sessions aimed at building skills needed to make good decisions about their SRH (57).

#### Support Clinicians

Eight studies supported clinicians by employing peer navigators that work in tandem with youth-friendly clinic providers to support referral and linkage to the YFHSs, recruiting new clinic staff, and establishing new roles within the clinics (40, 44, 51, 54–57, 59). One study also implemented an adolescent HIV risk screening tool to help clinicians identify and prioritize high-risk patients (44).

#### Provide Interactive Assistance

Six studies reported that ongoing supportive supervision was provided to the YFHS providers by external experts, consultants, or community stakeholders to address real-time implementation challenges and monitor implementation processes (40, 45, 49, 54, 55, 60).

#### Train and Educate Stakeholders

Sixteen studies (38–40, 42–45, 49, 51, 56, 57, 59, 60) employed a variety of training and educational strategies focused on five main concepts: (1) to provide factual SRH information for youth; (2) to promote changes in knowledge, attitudes, beliefs, and behaviors related to SRH among youth and other community members; (3) to deliver SRH services in ways that respond to the special needs of youths; (4) to appropriately provide STI diagnosis and treatment regimen for youths; and (5) to orient select youth to be advocates and change agents in their communities (i.e., peer educators). Ten studies reported the duration of the trainings and/or number of training sessions conducted, ranging from 1–5 days (38, 54–56, 60) to 1–4 weeks (40, 45, 49) or 12–22 (39, 57) training sessions.

### Implementation Outcomes

#### Overview

All 18 studies reported at least one implementation science outcome, ranging from 1 to 4 for each study (see Table 3). The most commonly reported Implementation science outcomes were adoption (*n* = 15, 83.3%), fidelity (*n* = 7, 38.9%), acceptability (*n* = 5, 27.8%) and penetration (*n* = 5, 27.8%).

**Table 3:** Implementation strategies used and implementation outcomes reported in the included studies.

**References**	**Implementation Strategies**		**Implementation Outcomes**								
	**ERIC Categories**	***n***	**Acceptability**	**Adoption**	**Appropriateness**	**Feasibility**	**Fidelity**	**Implementation cost**	**Penetration**	**Sustainability**	***n***
Aninanya et al. (38)	Engage consumers; Use evaluative and iterative strategies; Develop stakeholder interrelationships; Train and educate stakeholders.	4	x	x							2
Cowan et al. (39)	Use evaluative and iterative strategies; Adapt and tailor to the context; Train and educate stakeholders.	3					x				1
Fikree et al. (40); Fikree et al. (41)	Engage consumers; Use evaluative and iterative strategies; Support clinicians; Provide interactive assistance; Train and educate stakeholders.	5		x			x		x	x	4
Karim et al. (42)	Engage consumers; Change infrastructure; Develop stakeholder interrelationships; Train and educate stakeholders.	4		x					x		2
Kim et al. (43)	Engage consumers; Change infrastructure; Train and educate stakeholders.	3		x					x		2
Kose et al. (44)	Use evaluative and iterative strategies; Change infrastructure; Support clinicians; Train and educate stakeholders.	4		x					x		2
Larke et al. (45); Doyle et al. (46); Ross et al. (47); Terris-Prestholt et al. (48)	Engage consumers; Provide interactive assistance; Train and educate stakeholders.	3		x			x	x			3
Mathews et al. (49) and Mathews et al. (50)	Change infrastructure; Adapt and tailor to the context; Provide interactive assistance; Train and educate stakeholders.	4	x	x			x				3
Mbonye (51)	Change infrastructure; Support clinicians; Train and educate stakeholders.	3		x							1
Mmbaga et al. (52)	Use evaluative and iterative strategies; Adapt and tailor to the context; Develop stakeholder interrelationships.	3		x			x				2
Mmari et al. (53)	Engage consumers; Train and educate stakeholders.	2	x				x				2
O'Fallon et al. (54)	Engage consumers; Use evaluative and iterative strategies; Adapt and tailor to the context; Support clinicians; Provide interactive assistance; Train and educate stakeholders.	6		x						x	2
Ogu et al. (55)	Use evaluative and iterative strategies; Change infrastructure; Develop stakeholder interrelationships; Support clinicians; Provide interactive assistance; Train and educate stakeholders.	6		x	x						2
Okonofua et al. (56)	Engage consumers; Change infrastructure; Support clinicians; Train and educate stakeholders.	4		x					x		2
Rosenberg et al. (57)	Change infrastructure; Use financial strategies; Support clinicians; Train and educate stakeholders.	4		x			x				2
Smith et al. (58)	Change infrastructure.	1	x	x							2
Speizer et al. (59)	Engage consumers; Change infrastructure; Support clinicians; Train and educate stakeholders.	4		x							2
Wagner et al. (60)	Use evaluative and iterative strategies; Develop stakeholder interrelationships; Provide interactive assistance; Train and educate stakeholders.	4	x				x				2
Total			5	15	1	0	8	1	5	2	

#### Acceptability

Of the five studies (33, 38, 45, 50, 52) that reported on acceptability, four studies (38, 49, 58, 60) assessed youth satisfaction with services received and one study (60) measured the degree of job satisfaction among health providers in regards to the implementation climate. One study (53) measured community acceptance with the provision of reproductive health services for youth. Although there were variabilities in the acceptability measures, all five studies reported high ratings of acceptability toward the YFHS intervention implemented.

#### Adoption

Adoption was mainly measured as uptake and/or utilization of SRH services/commodities across the 15 studies (38, 40, 42–45, 49, 51, 52, 54–59), with studies reporting positive (38, 40, 42–44, 51, 55–57, 59), mixed (45, 52, 54), and no effect (49, 53) findings on the utilization and uptake of SRH services/commodities.

#### Appropriateness

Only one study (55) examined appropriateness, reporting significant improvement in the perceived importance of YFHS intervention among young people post-intervention.

#### Feasibility

There were no studies that measured feasibility of implementing YFHS.

#### Fidelity

Fidelity was reported in seven studies, which was mainly described as competency and/or adherence in delivering the interventions as intended. Training fidelity was assessed in one study by measuring changes in youth-friendliness among health providers (53). In terms of delivery fidelity, six studies reported using either self-reported measures by project staff or observation checklists by research team to assess the frequency (39, 50, 57), or presence or absence (41, 45, 52) of intervention components. All seven studies reported moderate to high levels of fidelity with only one study reporting operational constraints due to staff turnovers, absence of supportive supervision, and weak health system (41).

#### Costs

One study reported on the implementation cost and described that the cost of initial development of the intervention as well as the startup phase, were most substantial (48).

#### Penetration

Five studies examined penetration of YFHS, with only two of the studies (44, 56) reporting ratio-based metrics (i.e., the number of eligible participants who use services divided by the number of potential participants eligible to use services). Additional measures of penetration included reporting on the scale-up of the youth-friendly service delivery models across 182 health centers in four regions in Ethiopia (41) and high coverage of the YFHS outreach activities across multiple areas in the intervention group (42, 43).

#### Sustainability

Two studies included measures of sustainability. O'Fallon et al. examined post-intervention youth-friendly health service utilization and found that there was a decline in utilization after the youth outreach activities ended post-intervention (54). Whereas, Wagner et al. assessed whether there was a steady improvement in the primary and secondary outcomes over time and reported a sustained improvement in knowledge of HIV prevention and transmission throughout the intervention period (60).

## Discussion

Using the ERIC classification of implementation strategies and Proctor et al. taxonomy of implementation outcomes was helpful in understanding and comparing strategies used and outcomes achieved (26) across the 18 unique intervention studies. Although more recently, there has been increased interest in the use of modern methods (i.e., hybrid effectiveness-implementation trial design) in implementation science for testing implementation strategies and outcomes, our review identified significant knowledge gaps in the literature. Given that majority of the studies in our review did not identify as an implementation study, there were inconsistencies in the use of terminology and definitions related to implementation. The implementation strategies employed across the studies in our review were multifaceted, with an over-reliance on training and educating of stakeholders involved in the delivery of YFHS. Additionally, a vast majority of the included studies assessed early-stage implementation outcomes, such as adoption, acceptability and fidelity, whereas only a limited number of studies assessed later-stage implementation outcomes such as penetration, cost and sustainability (23).

The studies included in our review used a wide range of implementation strategies. The most frequently reported categories of implementation strategies were to train and educate stakeholders, change infrastructure and engage consumers. Whereas, implementation strategies such as providing interactive assistance, adapting and tailoring to local context and using evaluative and iterative strategies, were less frequently reported. Among the health service providers, several studies reported the use of training strategies to improve their knowledge, attitude and skills of healthcare workers to better respond to the needs of youth and this strategy was also found to be commonly used by others, even though the effects on clinical or service outcomes are inconsistent (5, 19, 62). Also, only a few studies reported to have trained select youth to identify and refer other youth to preventive services or provide psychosocial support and basic health education on SRH. In line with this, the limited engagement of youth in the design and implementation of YFHS across the studies, was surprising, given recent global efforts to enhance youth engagement in research beyond the typical beneficiary involvement (63, 64). Despite current consensus in the literature on the use of tailored approaches to implementation (30, 65), only four studies reported to have tailored the YFHS intervention to address local contextual factors to meet the local needs and organizational capabilities. Moreover, several frameworks for improving the provision and use of health services for youth, including the WHO quality of care framework (13, 66), emphasize the importance of tailoring health services to address the developmental needs of young people and the unique challenges they face. The limited use and reporting of strategies tailored to different implementation contexts and minimal engagement of youth across the lifespan of intervention research, may explain the limited implementation success (i.e., sustained use) of YFHS to date.

There was limited evidence comparing the effectiveness of different implementation strategies. In other words, no single strategy was identified as the main “driver” of change across the intervention studies. This may be due to the unique challenges and complexities of implementing an intervention in a low-resource setting which may require the use of different strategies in the absence of knowing what works. Similar to other studies (29, 67), we found no clear patterns between the number of strategies used and the magnitude of impact on the outcomes measured in the study. While researchers strongly advocate for the use of multifaceted strategies, we also found that the study by Smith et al. (58) reported the use of a single strategy and was considered successful on the basis of the implementation outcome measured in the study. A more recent review aimed at assessing whether multifaceted implementation strategies are more effective than single strategies, echo our findings with their insight that multifaceted strategies are not necessarily more effective than single strategies (68, 69). This may be indicative that the selection of strategies goes beyond quantity but more importantly, using a tailored approach to select strategies based on having a thorough understanding of context, including barriers and facilitators to implementation (29).

In addition, implementation outcomes were reported in all the included studies, with most measuring a narrow range of implementation outcomes. The lack of late-stage implementation outcomes (i.e., penetration, cost and sustainability) (23) in most studies was notable. Little consideration was given to the cost-effectiveness and other economic evaluations of the strategies, which makes large-scale investments in YFHS interventions unlikely due to the paucity of strong evidence of affordability and sustainability. Very few studies in our review reported sustainment-related outcomes, which suggest a potential limitation in the current implementation strategies, as it remains unclear as to what strategies facilitate or hinder sustainability outcomes (70). While the intended goal is to move from small scale, time-limited projects to larger scale, sustained programs to reach wider youth population, interestingly, outcome evaluations focused on scale-up (i.e., penetration) were rarely reported. Therefore, a key message from our review is the need for continued efforts in operationalizing, measuring, and reporting of implementation outcomes, paying close attention to the late-stage outcomes such as sustainability, penetration, and cost.

Although not the primary focus of this review, it is also worth mentioning that the youth-friendly health services were commonly delivered within the context of a health facility or clinic setting, with a few (3 out of the 18) studies that focused solely on out-of-facility service delivery approaches (49, 58, 59). Out-of-facility approaches aim to reach youth who may have limited access to a health facility or clinic and take the services to where they congregate or leave—schools, youth centers, on the street, etc. (15). Consistent with earlier reviews, evaluating the effectiveness of such approaches among adolescents (5, 15, 71), the three studies in our review that were undertaken in community-based settings (i.e., mobile clinics, schools, and youth centers) reported at least some positive, albeit generally weak, evidence on improving access to and uptake of SRH services (50, 58, 59). Further and rigorous implementation research is needed to better understand the effectiveness of out-of-facility approaches for delivering HIV and SRH services among young people in resource limited settings. Lastly, we found that majority of the included studies were conducted between 2015 to 2020, which might reflect the increasing recognition and commitment toward achieving the post-2015 development agenda on addressing adolescent sexual and reproductive health and rights (9, 72).

Our review has several strengths and limitations. This is one of the first systematic reviews to critically appraise published literature on implementation strategies used to enhance YFHS intervention in SSA. Further, this review uses bespoke compilation of implementation strategies (i.e., ERIC) and outcomes (Proctor et al.'s taxonomy). However, there is a potential risk that we may have omitted aspects not covered in the two categorizations given that different frameworks provide different lenses through which research questions/contexts are conceptualized. Further, despite the comprehensiveness of the ERIC categories, we experienced some challenges in regard to overlap between categories. Nevertheless, there were no discrepancies in data extraction between the two authors that conducted the validation process. The implementation outcomes that emerged from in the included studies were too sparse to draw strong conclusions about the strategies that promote or enhance the successful implementation and sustainment of YFHS interventions. Although we conducted a thorough search for relevant articles on youth-friendly sexual and reproductive health services, it is likely that we may have overlooked some articles based on our search strategy. For instance, given one of the aims of our review was to synthesize how Implementation strategies are used to enhance YFHS interventions in the academic literature and how these findings can inform future recommendations on reporting, we did not review gray literature. Therefore, we excluded unpublished and non-peer reviewed articles. We are aware of YFHS interventions in the field that have not been written up for publication (5), as such we acknowledge that our review is limited to fieldwide perspective on the academic literature.

## Conclusion

This review addresses a critical gap in the evidence-base and points to the need for more robust studies to test the effectiveness of implementation strategies. Consistent reporting of the steps followed, and adaptations made to tailor such services to local needs and capacity might contribute to standardizing and developing key set of strategies needed to enhance implementation and sustainment of YFHS interventions. Such research is needed to generate evidence that may in turn convince policy-makers of the value of scale-up.

## Data Availability Statement

The original contributions presented in the study are included in the article/Supplementary Material, further inquiries can be directed to the corresponding author/s.

## Author Contributions

CO-U and JI conceptualized the idea for this paper. CO-U produced the first draft of the manuscript. UN, SM, TG-B, DO, and OE reviewed the first draft, made edits, and provided comments on the manuscript. All authors have read and approved the final manuscript.

## Conflict of Interest

The authors declare that the research was conducted in the absence of any commercial or financial relationships that could be construed as a potential conflict of interest.

## Publisher's Note

All claims expressed in this article are solely those of the authors and do not necessarily represent those of their affiliated organizations, or those of the publisher, the editors and the reviewers. Any product that may be evaluated in this article, or claim that may be made by its manufacturer, is not guaranteed or endorsed by the publisher.
