# Supplementary material for: Implementation Strategies to Enhance Youth-Friendly Sexual and Reproductive Health Services in Sub-Saharan Africa: A Systematic Review
Source: Front Reprod Health. 2021 Aug 4;3:684081. doi: 10.3389/frph.2021.684081 (PMC9580831; doi:10.3389/frph.2021.684081)
Supplement: Supplementary file 1 [file Table_1.docx]

| **Supplementary Table 1. Table of evidence** | | | | | | | | |
| --- | --- | --- | --- | --- | --- | --- | --- | --- |
| **First author, year** | **Aim of the study** | **Country** | **Study Design** | **Outcome of Interest** | **Models for delivering YFS** | **Participants** | **Implementation Strategy** | **Key findings** |
| Aninanya et al., 2015 | To assess the effectiveness of a community-based ASRH intervention on Health Service Usage among young people | Ghana | Cluster-randomized trial: 26 communities randomly allocated to the intervention group (school-based SRH curriculum + out-of-school outreach + community mobilization + YFHS training) or the comparison group (community mobilization + YFHS training) | Health service usage within the past 12 months for STI management (i.e. diagnosis, treatment), HIV counseling and testing, antenatal care, perinatal services (e.g. delivery, postnatal care), and reported satisfaction with services received | Model 3 + Model 5 | 2,664 adolescents aged 15–17 | Develop stakeholder interrelationships: Conducted seminars and meetings with key community stakeholders.  Train and educate stakeholders: Trained health providers at the clinics on providing YFHS. Also, teachers were trained in SRH curriculum. Peer workers were trained and they supervised young people to provide SRH information and peer counseling.  Engage consumers: promote dialogue and interaction between adolescents and providers.  Use evaluative and iterative strategies: Assessed health facilities to address barriers to YFHS provision; Assessed satisfaction with services among adolescents. | The intervention resulted in a significant increase in adolescent usage of STI, antenatal and perinatal services. |
| Cowan et al., 2010 | To determine the effectiveness of a community-based, multi-component HIV and reproductive health intervention for young people | Zimbabwe | Cluster randomized trial that was conducted in 30 communities in seven districts | Rates of HIV‐1 and Herpes simplex virus type 2 (HSV‐2) | Model 3 + Model 5 | 4,684 participants, aged 18–22 | Adapt and tailor to context: MEMA kwa Vijana curriculum was adapted for use in Zimbabwe.  Train and educate stakeholders: Five-day residential youth‐friendly clinic staff training and a refresher training after 2 years.  Use evaluative and iterative strategies: Standards for YFS were developed by the nurses and were monitored and assessed based on these standards.  Engage consumers: Peer educators delivered interventions to youth, parents and community stakeholders. | Although there was no effect on the primary outcomes, HIV prevalence and HSV-2 prevalence, there was a modest improvement in knowledge related to STI acquisition. |
| Fikree et al., 2017; Fikree et al., 2020 | To evaluate the effect of training youth-friendly service providers (to counsel and provide all contraceptives) on uptake of contraceptives. | Ethiopia | Quasi-experiment: 10 intervention and 10 non-intervention YFS units | Long-acting reversible contraceptives (LARCs) and short acting methods uptake (pre-intervention to post-intervention phases) | Model 2 + Model 5 | 5,513 participants aged 15-24 years | Train and educate stakeholders: Peer educators were trained to dispel LARCs myths and misperception;  Project staffs provided LARCs competency-based training for the YFS providers. Provide interactive assistance: Utilized supportive supervision to address barriers faced by the YFS providers in real-time during routine monthly meetings.  Use evaluative and iterative strategies: Used qualitative assessments to understand the facilitators and barriers that influence the scaling up of the YFS delivery model.  Support Clinicians: Peer educators were assigned to refer prospective LARCs clients to the YFS unit within the public health clinic. | LARC uptake increased significantly in the intervention arm compared to the control arm. However, when the intervention was scaled to other clinics, only two out of eight clinics experienced an increase in LARC uptake overtime. Fidelity to the pilot model was operationally constrained. |
| Karim et al., 2009 | To evaluate the impact of the impact of the African Youth Alliance (AYA) program on the sexual behavior of young people | Uganda | Comparison of post-intervention survey data: self-reported exposure design and static group comparison design | Delay of sexual initiation; fewer sex partners; increase in condom use; consistent condom use; and increased use of contraceptives. | Model 3 + Model 5 | 3,176 Participants, aged 17-22 years | Develop stakeholder interrelationships: Established a mechanism within the clinic to ensure integration of AYA program components by sharing partners’ work plans and facilitating stakeholder engagement.  Engage consumers: promoted an enabling national and local environment for SRH programming through mass media campaigns and community engagement activities.  Train and educate stakeholders: Provided capacity-building opportunities for the institutions to utilize behavior change communications for SRH programming.  Change infrastructure: Established youth-friendly facilities (i.e. peer providers of services and institutionalizing SRH service curriculum). | AYA intervention had a positive impact on sexual behavior (condom use, consistent use of condoms, and contraceptive) among young females but not among young males; more than one-third of the study participants reported exposure to the AYA program in the intervention sites. |
| Kim et al., 2001 | To assess the effectiveness of media promotions on the adoption of behaviors that reduce the risk of pregnancy and STIs, including HIV among young people | Zimbabwe | Quasi-experimental design: five campaign sites (which was the experimental group) + Two other sites (which served as the comparison group) | Changes in reproductive health knowledge and attitudes; Service utilization | Model 3 + Model 5 | 1,426 at baseline + 1400 at follow-up; additional 700 participants to assess reach between ages 10-24 years | Engage consumers: Using a mix of communication channels (i.e. posters, leaflets, newsletters, radio programs, dramas), launch events and peer educators to increase SRH knowledge and use of SRH services;Established hotline at the youth center to disseminate campaign messages and answer queries from youths.  Train and educate stakeholders: Use of Train the trainer model whereby trained FP providers from each clinic trained other providers to counsel youths better.  Change infrastructure: Created designated clinics in the campaign areas as “youth-friendly". | There was a significant increase in contraceptive prevalence at the intervention site; Young people in the campaign sites were more likely to visit health centers or youth center when compared to participants in the comparison sites. |
| Kose et al., 2018 | To evaluate the effect of new intervention package on the uptake of HIV testing and linkage among adolescents | Kenya | Quasi-experimental study: a pre-and-post intervention study in 139 healthcare-facilities | HIV testing and linkage to care | Model 3+ Model 4 | 25,520 adolescents preintervention + 77,644 adolescents in postintervention between ages of 10-19 years | Train and educate stakeholders: Capacity building opportunities were provided to HCF providers.  Change infrastructure: Services were decentralized to lower-level facilities, such as dispensaries; Implemented extended hours for HCF.  Support clinicians: introduced adolescent HIV Risk screening tool to identify potential clients at the HCF; Additional HIV testing service providers were allocated.  Use evaluative and iterative strategies: Introduced program-specific monitoring and data collection tools at the HCFs. | A significant increase in the uptake of HIV testing was reported in the post-intervention compared to pre-intervention. Among individuals who tested positive, there was a significant increase in linkage to care and treatment services from the preintervention to postintervention |
| Larke et al., 2010; Doyle et al., 2010; Ross et al., 2007; Terris-Prestholt et al., 2006 | To assess the impact of an adolescent sexual health intervention on the use of health services by young people in Tanzania | Tanzania | Cluster-Randomized Trial: Randomization of 10 communities in the intervention arm and 10 communities in the control arm, with a total of 39 health facilities | HIV prevalence; HSV-2; Syphilis/Chlamydia/Gonorrhea prevalence; Condomless sex; Clinic use due to STI symptoms | Model 3 + Model 5 | 3,524 Attendees of intervention and 3,516 control schools surveyed at 3 years; 7,083 intervention and 6,731 control attendees surveyed at 9 years aged 12-24 years’ old | Train and educate stakeholders: Teacher-led, peer-assisted reproductive health education for youths; Refresher training for health providers at the clinic; Health providers were trained to provide YFS for SRH.  Engage consumers: Promotion of condom use by community youth leaders.  Provide interactive assistance: quarterly supervision visits by a supervisor trained in the provision of YFS. | A modest increase in young people's use of health services for STI related symptoms; No biological improvement on HIV and other STI prevalence. The initial development and startup costs were relatively high. |
| Mathews et al., 2016; Mathews et al., 2015 | To assess the effect of an after-school SRH education program and school health service on sexual debut and condom use among adolescents | South Africa | Cluster randomized controlled trial: conducted among grade eight students in 42 high schools | Consistent use of condom; Sexual debut and number of partners | Model 5 | 3,451 participants between the ages of 12 to 19 years | Adapt and tailor to context: The program was pilot-tested among a subset sample over 3 years prior to full implementation.  Train and educate stakeholders: Implemented a 21-session educational program delivered by trained facilitators in after-school clubs.  Change infrastructure: School health service was delivered by a nurse from the nearest public clinic within the school premises, once a week during after school hours.  Provide interactive assistance: The trained facilitators received weekly supportive supervision. | No differences observed between the intervention and control arm for the primary outcomes (sexual debut, condom use and a number of sexual partners); sub-optimal exposure to the after-school education sessions and school health service; Participants in the intervention group had increased condom and HIV/AIDS knowledge; High acceptability of the intervention. |
| Mbonye et al., 2003 | To evaluate the impact of a pilot adolescent health service intervention on service utilization | Uganda | Quasi-experimental, non-randomized control group with four intervention clinics and four control clinics | Uptake of FP and STI management services | Model 3 | 128 participants between the ages of 10-19 years | Train and Educate: Trained healthcare workers in communication and counseling adolescents.  Support clinicians: Assigned designated youth-friendly providers in the clinic.  Change infrastructure: Health centers were equipped with basic supplies and recreational services introduced to engage youths. | The overall percent of adolescents who utilized the pilot clinic for the management of STI and family planning services were significantly greater compared to the control clinics. |
| Mmbaga et al., 2017 | To assess the effect of an after-school SRH education program and school health service on sexual initiation and condom use among adolescents | Tanzania | Cluster randomized controlled trial: conducted in 38 public primary schools | Sex initiation; condom acquisition and use | Model 1 + Model 5 | 5,091 participants between the ages of 12-14 years | Adapt and tailor to context: Intervention was adapted based on the findings from the formative phase; 3 peer-led after-school lessons and 6 teacher-led lessons were integrated into the primary school science curriculum.  Develop stakeholder  interrelationships: Promote collaboration between schools and youth-friendly health services to increase access to SRH information and services. Use evaluative and iterative strategies: Feedback sessions following youth-friendly clinic visits. | A significant effect on condom use behavior was observed among male adolescents in the intervention arm; overall positive effect in reducing sexual initiation among males and females in the intervention arm. |
| Mmari KN et al., 2003 | To assess the effectiveness of three youth-friendly services (YFS) projects on service utilization | Zambia | Longitudinal Service statistics: 3 intervention groups (8 clinics in total that offered YFSs) + 1 control group (2 clinics with no YFS component) | Youth-friendliness of the services, community acceptance and service use | Model 3 + Model 5 | 15-24 years | Train and educate stakeholders: Train health providers and peer educators to better communicate with young people.  Engage consumers: Community sensitization activities. The youths selected peer educators. | No substantial increase in service utilization in the clinics that had implemented YFS projects versus control clinics |
| O'Fallon et al., 2020 | To evaluate the effect of a YFHS training package on FP uptake ((IUD, implant, oral contraceptive pill, injectable, condoms, or counseling only) and service utilization | Malawi | Longitudinal service statistics among 39 YFHS training intervention clinics | Utilization of FP | Model 3 | 72-2278 per quarter between the ages of 15-24 years | Use evaluative and iterative strategies: Applied quality assurance standards for all voluntary FP service delivery programs.  Provide interactive assistance: Program staffs provided ongoing supportive supervision to health providers implementing YFHS in their clinics.  Train and educate stakeholders: Conducted annual YFHS training. Engage consumers: Utilized interpersonal communication agents to engage youths in a one-on-one session; Conducted awareness activities and informational campaigns among local communities and religious leaders.  Support Clinicians: Communication agents facilitated referrals to the YFHS. Adapt and tailor to context: Trainings were tailored to fit the local context and needs of the health providers. | Observed positive trends in the utilization of FP services among the young people at every quarter during implementation. However, there was a decline after youth outreach activities ended. |
| Ogu et al., 2018 | To determine the influence of integrating youth-friendly health services (YFHS) into existing primary health care facilities in rural regions | Nigeria | Quasi-experiment design: Two primary health facilities located in two geographically distinct communities | Self-reported utilization of YFHS; perception of availability, affordability and barriers to utilization | Model 3 | 465 participants for preintervention and 558 participants for postintervention, 10-24 years | Develop stakeholder interrelationships: Community-level stakeholder engagement activities to foster community ownership.  Train and educate stakeholders: Capacity building training for healthcare workers focused on strengthening technical skills and social competencies.  Change infrastructure: Identification and designation of a physical space for YFHS in the healthcare facility. Support clinicians: human resource mobilization.  Use evaluative and iterative strategies: Conducted gap analysis and needs assessment.  Provide interactive assistance: Convened technical working group made-up of community key stakeholders and youth representatives that monitored the project. | Significant increase in the utilization of YFHS from post-intervention compared to pre-intervention. |
| Okonofua et al., 2003 | To evaluate the impact of an intervention to improve STI treatment-seeking behaviors and treatment provision | Nigeria | Randomized controlled trial: 1 intervention group (total of 4 schools) + 2 control groups (total of 8 schools) | Knowledge of STI symptoms; condom use; treatment-seeking behavior; STI prevalence | Model 3 + model 5 | 1,896 participants for preintervention and 1,885 participants for postintervention 14 and 18 years. | Engage consumers: Adolescents identified private providers (practitioners, patent medicine dealers, pharmacists) frequently used by youths within their neighborhood.  Train and educate stakeholders: Convened health club and trained members as a peer educator to provide counseling to other adolescents; Trained the private providers on STI diagnosis and treatment regimen.  Change infrastructure: Established a reproductive health club in each school to promote SRH information.  Support clinicians: The trained clinics were certified as adolescent-friendly health providers. | Significant increase in condom use among males and females in the intervention group compared to the two control groups. The proportion of youth who sought treatment for symptoms of STIs from the YF health providers increased in the intervention group. The prevalence of STI symptoms reduced significantly in the intervention compared to the control groups. |
| Rosenberg et al., 2018 | To assess whether a YFHS delivery model has impact on HIV and SRH service utilization | Malawi | Quasi-experimental prospective cohort study: Compared 4 models (standard of care, YFHS, YFHS + Behavioral Intervention (BI), YFHS + BI + conditional cash transfer(CCT)) in 4 health centers | HIV testing uptake, condom uptake, contraception uptake, STI uptake | Model 2 | 1,000 participants (250 AGYW per health center) between the ages of 15-24 years | Train and educate stakeholders: Healthcare providers received Youth-friendly trainings and sensitizations; Conduced small-interactive group sessions for youths that addressed communication skills, HIV and SRH information.  Support clinicians: Youth peer educators were utilized to offer health education and support clinic navigation.  Use financial strategies: Participants received a monthly CCT for attending each BI session.  Change infrastructure: Created a dedicated youth-focused space where SRH services were provided and Extended hours of operation. | There was considerable higher uptake of HIV test, condom use, contraception use and STI-related visit in the 3-intervention group compared to the standard of care group. |
| Smith et al., 2019 | To evaluate the acceptability of adolescent and youth-friendly mobile SRH services | South Africa | Cross-sectional comparison of a mobile clinic to four conventional government clinics | Acceptability and usage of Mobile clinic; HIV outcomes | Model 4 | 303 participants between the ages of 16-24 years | Change infrastructure: Implemented a mobile clinic (i.e. mobile van) to provide SRH services in a central area of a community. | Overall acceptability of the mobile clinic was high. The mobile clinic yielded more HIV diagnosis compared to the conventional clinics. |
| Speizer et al., 2004 | To evaluate the impact of youth center on contraceptive use and determining the appropriateness of a youth center as a service delivery model. | Togo | Before and after, panel study design | Contraceptive uptake | Model 5 | 2,083 youth between the ages of 10-24 years; 1,679 youth recruited for first follow-up period; 1,332 youth recruited for second follow-up period. | Change infrastructure: Developed SRH services within youth centers. Engage consumers: Youth leaders in the community worked with project staffs to help inform the youth center planning process and ensure that youth needs were addressed.  Train and educate stakeholders: Trained clinic staff in reproductive health service delivery for youth.  Support clinicians: Trained peer educators to facilitate outreach and referral activities in the community. | Slight increase in the use of youth centers for clinical services over the follow-up period. |
| Wagner et al., 2017 | To determine whether a Continuous quality improvement intervention improves the quality of adolescent and young people testing services | Kenya | Time-series and pre/post-analytic approaches within two clinics | The satisfaction of services; intent to return, knowledge of HIV prevention and transmission | Model 3 + Model 5 | 172 participants recruited at baseline and 702 participants during the intervention period between the ages of 14 - 24 years | Develop stakeholder interrelationships: leadership teams were formed.  Train and educate stakeholders: In-person tannings were conducted on the model for improvement; the supervisors at the clinic received training on constructive feedback.  Provide interactive assistance: The healthcare workers received coaching to plan for QI intervention by project staff. Use evaluative and iterative strategies: HCW held weekly meetings to discuss change concepts. | Substantial improvement in the intent to retest at one clinic and Knowledge of HIV prevention and transmission. High satisfaction with visit experience, which was also sustained throughout the study period. |
